# Hierarchical assembly and environmental enhancement of bacterial ice nucleators

**DOI:** 10.1073/pnas.2409283121

**Published:** 2024-10-17

**Authors:** Galit Renzer, Ingrid de Almeida Ribeiro, Hao-Bo Guo, Janine Fröhlich-Nowoisky, Rajiv J. Berry, Mischa Bonn, Valeria Molinero, Konrad Meister

**Affiliations:** ^a^Department of Molecular Spectroscopy, Max Planck Institute for Polymer Research, Mainz 55128, Germany; ^b^Department of Chemistry, The University of Utah, Salt Lake City, UT 84112-0850; ^c^Materials and Manufacturing Directorate, Air Force Research Laboratory, Wright-Patterson Air Force Base, Dayton, OH 45433; ^d^Department Multiphase Chemistry, Max Planck Institute for Chemistry, Mainz 55128, Germany; ^e^Department of Chemistry and Biochemistry, Boise State University, Boise, ID 83725

**Keywords:** ice-nucleating proteins, protein assembly, bacteria, heterogeneous ice nucleation

## Abstract

Bacterial ice nucleating proteins (INPs) are exceptionally effective in promoting the kinetically hindered transition of water to ice. Their efficiency relies on the assembly of INPs into large functional aggregates, with the size of ice nucleation sites determining activity. Experimental freezing spectra have revealed two distinct, defined aggregate sizes, typically classified as class A and C ice nucleators (INs). Despite the importance of INPs and years of extensive research, the precise number of INPs forming the two aggregate classes, and their assembly mechanism have remained enigmatic. Here, we report that bacterial ice nucleation activity emerges from more than two prevailing aggregate species and identify the specific number of INPs responsible for distinct crystallization temperatures. We find that INP dimers constitute class C INs, tetramers class B INs, and hexamers and larger multimers are responsible for the most efficient class A activity. We propose a hierarchical assembly mechanism based on tyrosine interactions for dimers, and electrostatic interactions between INP dimers to produce larger aggregates. This assembly is membrane-assisted: Increasing the bacterial outer membrane fluidity decreases the population of the larger aggregates, while preserving the dimers. Inversely, Dulbecco’s Phosphate-Buffered Saline buffer increases the population of multimeric class A and B aggregates 200-fold and endows the bacteria with enhanced stability toward repeated freeze-thaw cycles. Our analysis suggests that the enhancement results from the better alignment of dimers in the negatively charged outer membrane, due to screening of their electrostatic repulsion. This demonstrates significant enhancement of the most potent bacterial INs.

## Significance

Fifty years ago, bacteria were identified as remarkable ice nucleators that enable water freezing close to 0 °C. Their record-holding activity is key for frost damage to crops and snow making. While it is known that bacterial freezing relies on the assembly of ice nucleating proteins (INPs), details of the underlying assembly mechanism and structures remained elusive. Here, we elucidate the size of the INP multimers responsible for superior ice nucleation and their formation process. We unravel a hierarchical assembly mechanism that explains the distinct ice nucleation temperatures of bacteria and their sensitivity to environmental factors. We demonstrate enhancement of the ice nucleation potency of the bacteria by controlling the pH and ionic content, expanding their potential use in freezing applications.

Bacterial ice nucleators (INs) are highly efficient in initiating ice crystallization at high subzero temperatures. Their remarkable control of the crystallization process is based on specialized ice-nucleating proteins (INPs) that are anchored to the outer membrane of the bacterial cell wall and are proposed to form large functional aggregates (1–3). The most extensively studied bacterial INs are *Pseudomonas syringae*, which enable ice formation at temperatures up to −2 °C (4, 5). As plant pathogens, they use their freezing capabilities to provoke frost injuries to plant tissues, allowing them to access the plant’s nutrients (6). Together with their widespread distribution (7–9), this makes *P. syringae* a significant contributor to frost damage in the biosphere, causing agricultural losses through crop devastation (10). Bacterial INs have further been detected in rain, hail, and snow, pointing to a role in atmospheric freezing processes, influencing the hydrological cycle and the overall balance of Earth’s climate (11–14). Due to their superior freezing efficiency, bacterial INs are widely used for artificial snowmaking and are raising attention for potential use in cryopreservation, anti-icing surfaces, and novel freezing technologies (15–17). Despite their importance and the extensive research over the past years (1–3, 9, 18–26), several questions remain unanswered regarding their mode of action and the possibility of enhancing their ice nucleation efficiency and resistance to freeze-thaw cycles. These are the questions we address in the present study.

The principal modus operandi of INPs is to provide a surface that strongly binds to ice, thus decreasing the free energy barrier for ice nucleation (3). The strong binding is supported by the ordering of water at the ice-binding surfaces (IBSs) of the INP (18–20). The general structure of bacterial INPs is subdivided into three domains (26), with the central repeating domain (CRD) comprising the majority of the structure. The CRD contains the active site and is formed by a variable number of tandem repeats of a highly conserved 16-residue sequence (18). The N-terminal domain is involved in anchoring the protein to the membrane, and the C-terminal domain is proposed to have a capping function to stabilize the protein structure (20). However, the structural characterization of INPs, remains challenging due to their large size (~130 kDa) and localization in the outer membrane (OM). Several theoretical studies predicted β-solenoid folds for the CRD of INPs, where the active site consists of long arrays of threonine-x-threonine (TxT) motifs located on one side and serine-lysine-threonine (SLT) motifs located on the opposite side of the flat solenoid structure (20–23, 27).

While an INP structure provides water-organizing motifs of high repetitiveness, the ice-binding area of monomeric INPs is insufficient to achieve high ice nucleation efficiencies. To accomplish freezing close to 0 °C, bacterial INs critically depend on their ability to form large INP multimers (2, 3, 24, 28, 29). A distribution of sizes of ice nucleation sites is further required to explain the typically broad freezing range from −2 to −12 °C. Freezing assays revealed that bacterial INs are active in particular temperature regions, which led to the categorization of INs into three distinct classes (30). Class A comprises the most efficient INs active at temperatures above −4.4 °C, class B IN are active between −4.4 and −7.6 °C, and class C IN active below −7.6 °C (30). Although there is agreement that Class C INs correspond to small INPs aggregates, and class A INs to larger INP assemblies, little is known about the definite size and numbers of INPs involved in these multimers and their assembly mechanism (2, 23, 24). Simulations and nucleation theory have addressed the size and assembly pattern of aggregated INPs (3). However, quantitative predictions are highly sensitive to the actual dimension of the protein binding surface, distance between monomers, alignment between the INPs, as well as strength of protein–ice interactions (3). Furthermore, an intact bacterial membrane has been shown to be required for functional aggregation of INPs (31–33). Removal of the membrane abolishes class A IN activity, and the addition of membrane lipids fails to restore it (31). Membrane fluidity disrupting agents have been shown to decrease class A activity (4, 32, 34), indicating that the membrane is not only a matrix for INP assembly but also has a decisive role in functional aggregation.

**Fig. 1.**
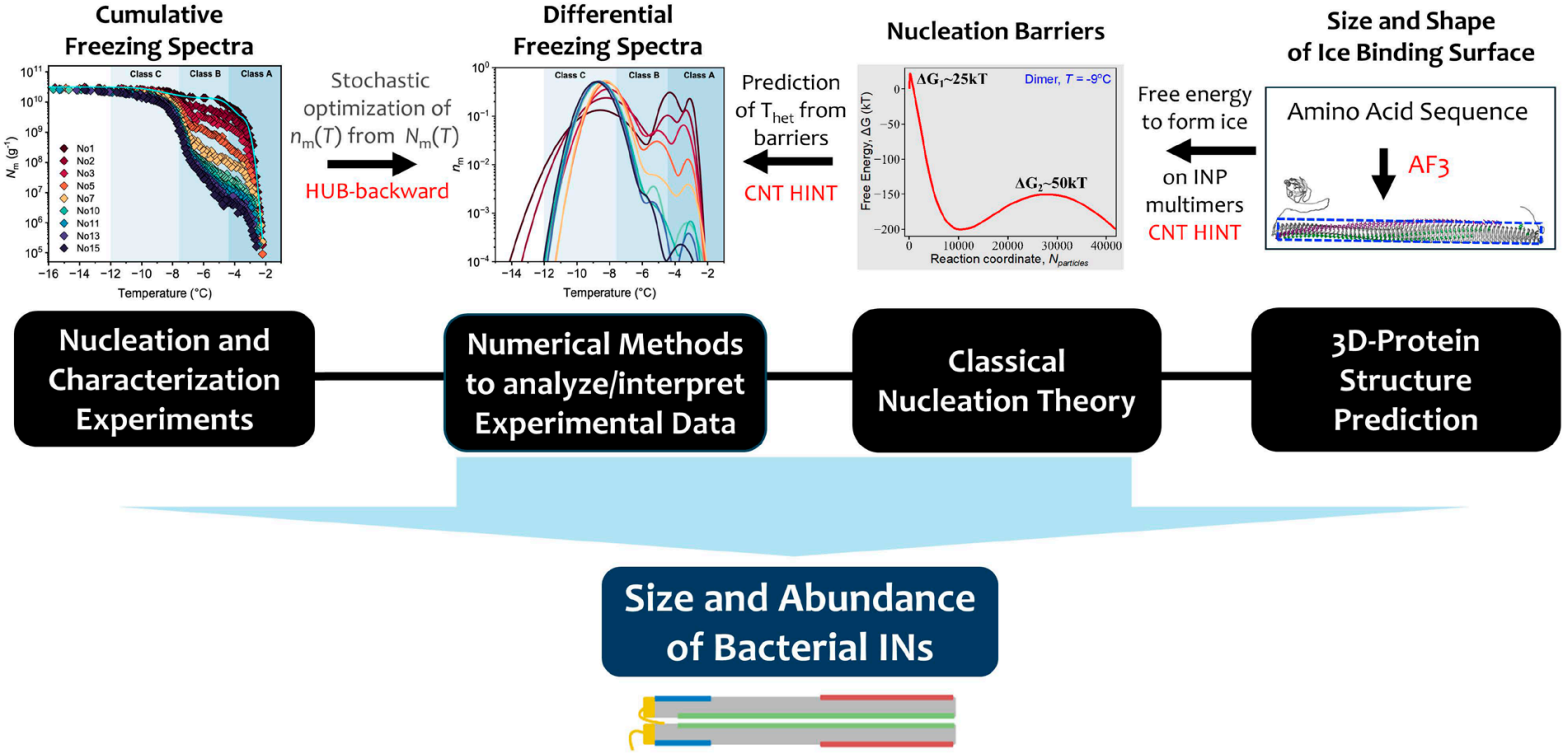
Schematic overview of our approach to elucidate the size and prevalence of bacterial INs. Starting with the cumulative freezing spectra (*N*_m_) obtained from our ice nucleation measurements, we employ a stochastic optimization method (the HUB-backward algorithm) (35) to determine the differential freezing spectra *n*_m_(*T*) and represent them as a linear combination of IN subpopulations. We utilize a combination of modeling and theory to interpret the physical meaning of these IN subpopulations in terms of the sizes of INP aggregates. From *Right* to *Left* in the figure, we first use AlphaFold (36) to predict the structure of the bacterial INP from its sequence (18). From the 3D-protein structure, we extract the width and length of the β-helix that constitutes its IBS (21, 22). We then compute the dimensions of the INP N-mers, assuming they have the same length and N-times the width of the IBS of a single INP. Next, we compute the free energy of nucleating and growing ice on the IBS of each N-mer using the HINT algorithm (3). The free energy profiles reveal two barriers: the first to nucleate ice on the protein and the second to grow the ice out of the protein into the surrounding solution. Using the magnitude of the larger barrier and the formalism of CNT implemented in the HINT algorithm, we determine the temperature of heterogeneous nucleation (*T*_het_) for the INP N-mers. We then compare this *T*_het_ with the *T*_het_ of the mode of the subpopulations in *n*_m_(*T*) determined from the experimental (*N*_m_). We find excellent agreement between the *T*_het_ for the class C peak and the one predicted for the INP dimer.

Here, we combine ice nucleation and membrane fluidity experiments, numerical modeling, AI-based protein structure prediction, and nucleation theory to elucidate the number of proteins in the IN of *P. syringae* as shown in a schematic overview in Fig. 1. We then propose a hierarchical mechanism for their assembly, confirm the key role of the outer membranes in the stability of larger aggregates, and demonstrate that the right combination of pH and ions in the ice nucleating solution preserves the large INs against disassembly in freeze-thawing cycles and -most surprisingly- promotes their assembly. Using these insights, we make Snomax®as potent an ice nucleation agent as the most potent strains of live *P. syringae*.

## Results and Discussion

### Freeze-Thaw Cycles Result in the Disassembly of Large INP Aggregates into Smaller Ones.

**Fig. 2.**
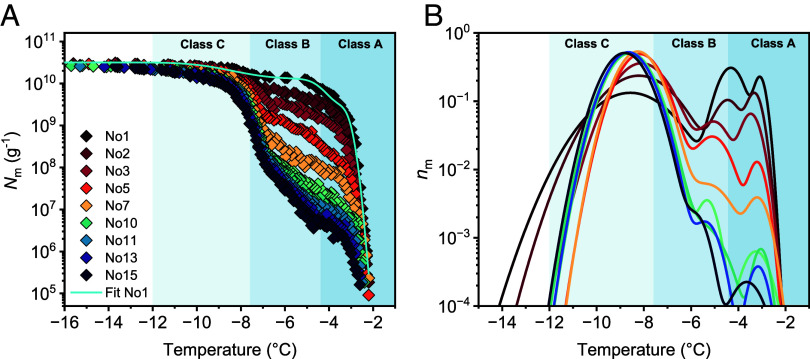
Freezing experiments of aqueous samples containing bacterial INs from *P. syringae* after repetitive freeze-thaw cycles. (*A*) Cumulative number of INs per unit mass of *P. syringae* (*N*_m_). The cyan line represents the optimized solution obtained through the HUB method, assuming that the differential spectrum is a linear combination of three Gaussian subpopulations. (*B*) Normalized distribution function that represents the corresponding differential freezing spectra *n*_m_(*T*).

Fig. 2*A* shows the cumulative freezing spectra of bacteria from the strain *P. syringae* Cit7, subjected to consecutive freeze-thaw cycles. The initial freezing spectrum of a 10-fold dilution series of alive *P. syringae* in water with an initial concentration of 0.1 mg/mL displays a wide range of freezing temperatures with maximum ice nucleation activity up to −2 °C. The freezing spectrum shows a strong increase in the cumulative IN concentration *N*_m_(*T*) per unit mass of bacteria at −2.4 °C and a second increase at −7.5 °C, with plateaus between −5 and −7 °C and below −9 °C. The two steep increases in the freezing spectrum indicate that the activity of *P. syringae* originates from IN species with different nucleation efficiencies. In contrast, plateaus indicate few active INs at these temperature ranges. Based on their nucleation temperatures, we assign the IN species to class A (−2.4 °C) and C (−7.5 °C) INs. We did not observe class B INs in the cumulative freezing spectra, in agreement with previous studies (2, 37–39).

The ice nucleation activity shows a consistent trend when the bacteria undergo repetitive freeze-thaw cycles. Class A INs exhibit progressive degradation, leading to a lower cumulative concentration, as well as a shift to lower temperatures. In contrast, *N*_m_(*T*) of class C INs increases throughout the cycles. Since freeze-thaw cycles do not cause chemical changes in the INP aggregate structures, this alteration in the ice nucleation activity must originate from physical effects. These observations align with previous findings which showed that bacterial INs with high freezing efficiencies are unstable toward changing environmental conditions, whereas IN active at lower temperatures remain stable (28, 30, 38).

To systematically investigate the changes in the freezing spectra for every cycle, we analyzed the underlying distribution of heterogeneous ice nucleation temperatures (Fig. 2*B*). The differential freezing spectra are derived from the cumulative spectra by using the Heterogeneous Underlying-Based (HUB) method (35). This analysis implements a stochastic optimization procedure that fits the experimentally obtained cumulative spectra with a linear combination of Gaussian subpopulations. The resulting computed differential spectra that reproduce the distribution of ice nucleation temperatures allow for a characterization of the underlying IN classes.

We find that fitting the spectra with a combination of two subpopulations based on the assignment of two distinct IN classes does not match the initial freezing spectrum of *P. syringae* and exhibits a discrepancy between −3.8 and −7.3 °C and a mean squared error (MSE) of 0.53% (for details, see *SI Appendix*, Fig. S1). A more accurate solution is achieved by considering three subpopulations, which lowers MSE to 0.1%, a factor of five improvement for 1.5 times the number of fit parameters. The resulting differential spectrum reveals that 49% of the IN nucleate ice at −8.6 ± 1.5 °C, 39% are active at −4.3 ± 0.5 °C, and 12% are active at −3.1 ± 0.2 °C. We assign the subpopulation active at −8.6 °C to class C. The other subpopulations are assigned to class A INs, since both modes fall into the class A regime (30). This implies that the ice nucleation activity of *P. syringae* relies on more than two IN populations and that class A consists of more than one aggregate species.

The HUB analysis also reveals that class C INs increase from 49 to 99.6% within ten freeze-thaw cycles, while the class A subpopulations decline over hundred-fold, from 39 to 0.3% and from 12% to less than 0.1%. Throughout these changes in the IN populations, the total cumulative concentration of INs per mass unit remains constant, highlighting that no INs are lost. These results prove that class A INs are not destroyed, but rather transformed into class C INs through repetitive freezing. Moreover, we observe an additional temperature shift for both class A subpopulations to lower temperatures. The IN subpopulation active at −4.3 °C changes to −5.3 °C, shifting from class A into class B regime. The HUB analysis shows that class C INs are the most stable IN population, since their fraction does not decrease over time and the total IN number remains constant. Although class B INs are not in the original cumulative freezing spectra, the distribution analysis confirms its presence after several freeze-thaw cycles. We conclude that the higher-ordered class A INP aggregates are destabilized during repetitive freezing and disassemble into smaller INP aggregates with lower ice nucleation temperatures.

### Class C Are INP Dimers, B Are Tetramers, and A Are Hexamers and Larger Aggregates.

**Fig. 3.**
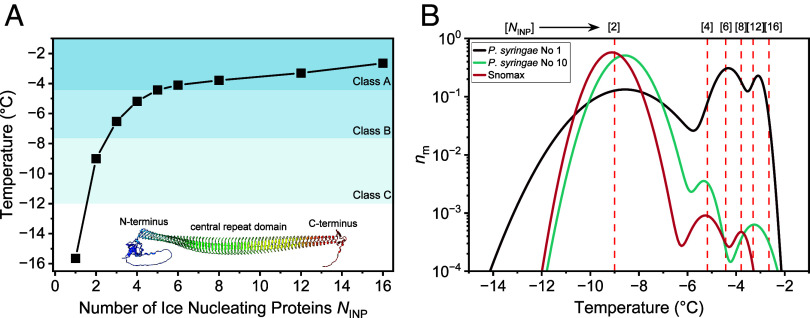
Quantification of INPs for aggregates responsible for the observed distinct IN subpopulations. (*A*) Ice nucleation temperatures as a function of the number of INPs *N*_INP_ from *P. syringae* predicted by CNT implemented in the HINT algorithm (*SI Appendix,* Table S2). Black squares show the freezing temperatures of rectangular surfaces derived from parallel aligned INP monomers considering a monomer with an IBS of 3.4 nm width and 30 nm length (from the structure of the monomer predicted by Alphafold, shown as *Inset*). The freezing temperature of ice increases with the number of protein monomers in the INP aggregates. The blue shadowed regions indicate the typical temperature ranges of class A, B, and C. (*B*) Normalized distribution function of bacterial samples representing the corresponding differential freezing spectrum *n*_m_(T). Vertical lines are based on the HINT predictions and indicate the freezing temperatures of different aggregates. The number in squared brackets describes the *N*_INP_ of the aggregate.

To determine the number *N*_INP_ of INPs required for the formation of aggregates promoting ice formation at the observed freezing temperatures, we utilize the heterogeneous ice nucleation temperature (HINT) algorithm (3). HINT uses experimental data for water to compute the free energy barriers to form ice on surfaces of finite size and propagate the growth into the surrounding solution, and then employs classical nucleation theory (CNT) to predict from these barriers the freezing temperature (Fig. 1) (3). We assume for the HINT calculation that the IBS is flat and the distance between monomers remains constant. The *Inset* of Fig. 3*A* shows the predicted AlphaFold structure of the INP of *P. syringae* (40). The protein fold is predicted with high confidence level (*SI Appendix*, Fig. S2) and reveals a length of ~30 nm and a maximum width *d* of the CRD of about 3.4 nm. Previous simulations showed that increasing the length of the water-organizing β-helix of INPs does not have a significant effect on the corresponding ice nucleation temperature, whereas expanding the width of the system by parallel alignment of INPs results in enhanced efficiency (3). Fig. 3*A* shows the predicted ice nucleation temperatures of the side-by-side INP aggregates, together with the ranges for classes A, B, and C.

Fig. 3*B* presents an overlay of the distribution of populations of IN in *P. syringae* on the 1st and 10th freeze-thaw-cycle, together with the predicted ice nucleation temperatures *T*_het_ as a function of the number of side-by-side INPs at the ice-binding site. By comparing the results of HINT with the distribution analysis of the freeze-thaw experiments, we obtain a correlation between INP aggregate sizes and the corresponding temperature modes of the subpopulations. We find no INP monomers or trimers in the bacteria: Class C activity corresponds to the INP dimer, confirming the assignment of previous studies (23, 41). The efficiency of such dimers relies on the equal ice nucleation ability of the two sides of the INP. Our analysis reveals that a minimum of six INPs are required to reach the temperature range of class A INs.

We conclude that in the initial freezing spectrum of *P. syringae* the class C INs originate from INP dimers, the class A INs with lower efficiency (−4.3 °C) from hexamers, and the most efficient class A INs (−3.1 °C) from multimers containing at least 12 INPs. No significant changes occur to the class C dimers upon freeze-thaw cycling, whereas the other subpopulations change dramatically along these cycles. Fig. 3*B* supports that the class A IN subpopulation corresponding to hexamers disassembles to produce tetramers, which we assign as class B INs, resulting in the disappearance of the larger multimeric aggregates of Class A INs from the freezing spectrum.

To generalize our findings, we analyzed the widely used sample Snomax® (Fig. 3*B* and *SI Appendix*, Fig. S3) (42), which consists of inactivated bacteria from *P. syringae*. Similar to alive bacteria, the inactivated bacteria show class A and class C INs (*SI Appendix*, Fig. S3), which we assign to INP multimers and the INP dimer, respectively. The distribution analysis of Snomax further confirms the existence of class B INs originating from INP tetramers.

### Size of Aggregates Is Consistent with a Dimer-Based Assembly Mechanism Driven by Electrostatics.

The assignment of even-number INP aggregates (dimers, tetramers, octamers, etc.) defies previous expectations of a continuum of sizes. It elicits the question of what determines the proportion of INP aggregates and their assembly mechanism.

**Fig. 4.**
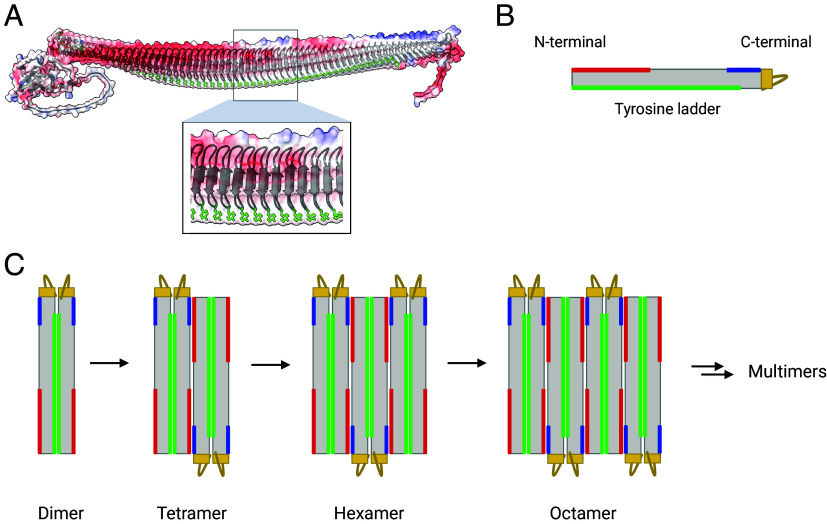
Prediction of Assembly of INPs into functional aggregates. (*A*, *B*) Electrostatic surface map of the INP monomer. Positively charged residues (blue) are clustered on one site closer to the C terminus, whereas negatively charged residues (red) are found closer to the N terminus. (*C*) INP assembly mechanism into dimers, tetramers, hexamers, octamers, and higher-order multimers. Dimer formation is mediated through the stacking of the solvent-exposed tyrosine ladders (green areas) between adjacent monomers and is assisted by the C-terminal tail (yellow) which likely acts as a cap. The tetramer, hexamer, and octamer formation are mediated through electrostatic interactions by outward-facing positively and negatively charged residues found on opposing ends of the β-solenoids opposite to the tyrosine ladder. Octamers can be formed by electrostatic interactions between a tetramer and two dimers or between two tetramer units.

We interpret that the dimerization of INPs is due to the stacking of the highly conserved tyrosine ladders (21), which create a surface where the ice-making motifs on the two sides of the β-helix align to expand the width of the combined nucleation site, and that the assembly of the larger aggregates is driven by electrostatic interactions. Fig. 4*A* shows the electrostatic surface model of the INP, which we obtained using AlphaFold. The INP model shows a clear pattern of charged residues. A large number of negative residues are located close to the N-terminal domain of the solenoid, whereas several positively charged residues are found close to the C-terminal domain. In addition, the tail of the C-terminal domain also contains charged residues. Our calculations with Alphafold version 2.3.2 (43) predict that the *P. syringae* INP dimer and higher multimeric aggregates form twisted amyloid structures that bury the ice-nucleating protein surfaces from water (*SI Appendix*, Fig. S4). Those structures are inconsistent with the high ice nucleation temperatures of the bacteria.

We propose that INP dimers assemble into tetramers through electrostatic interactions by the outward-facing positively and negatively charged residues found on opposing ends of the β-solenoids and opposite to the tyrosine ladder (Fig. 4*B*). The INP tetramer formation is assisted by the disordered charged tail of the C-terminal domain, which acts as a cap and provides structural stability. The essential function of the C-terminal domain in the proposed dimer and tetramer assembly mechanism is in line with previous reports that showed that deletion of the C-terminal domain eliminated all activity (20, 41, 44). Capping structures are further known to be essential for the stability of β-solenoids, and in their absence, the solenoids tend to unravel or form amyloid fibrils (45).

### The Bacterial Outer Membrane Is Key for the Assembly of Dimers into Larger Aggregates.

Having established the size of the INP aggregates in the bacteria, we focus on the role of the outer membrane (OM) in the functional INP assembly. It has previously been shown that aggregation of class C bacterial INPs can occur in solution (23), but the OM is needed to produce the most active INs. To probe the role of the OM on the stability of bacterial INs, we measure ice nucleation by Snomax as a function of treatment temperature, in the presence of membrane fluidizing agents, at low pH, and in the absence and presence of ions. By focusing on inactivated bacteria, we can exclude physiological factors such as protein synthesis, or homeoviscous adaptation, enabling us to single out the physical impact of environmental changes and cosolutes on OM fluidity and their impact on ice nucleation.

**Fig. 5.**
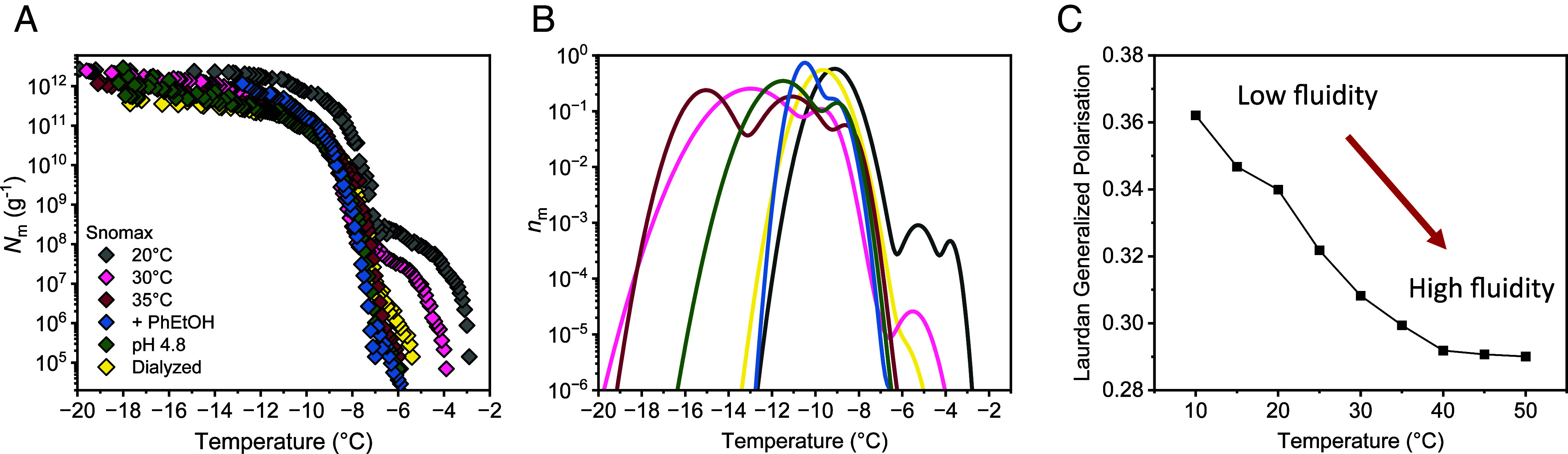
Freezing experiments of aqueous samples containing bacterial IN from Snomax after targeting the membrane fluidity through temperature, membrane fluidizing agents, the pH level, and ion removal through dialysis. (*A*) Cumulative number of INs per unit mass of Snomax. (*B*) Normalized distribution functions that represent the corresponding differential freezing spectra. *SI Appendix*, Figs. S7–S9 and Table S3 present the fits of the *N*_m_ of the heat- and pH-treated Snomax with various numbers of subpopulations. The existence of subpeaks in class C results from the fit with a small number of Gaussian subpopulations; we interpret that class C is only dimers, with the broadening resulting from a distribution of distances between the INP. (*C*) Generalized polarization values as a function of temperature of the membrane dye Laurdan incorporated into the membrane of Snomax. The fluorescence intensity was measured at emission wavelengths of 440 and 490 nm (*SI Appendix*, Fig. S6).

Fig. 5*A* shows the cumulative freezing spectra of Snomax as a function of changing environmental conditions. We find that when Snomax is subjected to warm temperatures prior to ice nucleation activity measurements, class A INs disintegrate and the distribution of class C broadens, as evidenced in the freezing spectra and the corresponding distribution functions (Fig. 5*B*). This effect becomes more pronounced at higher temperatures, with class A and B INs completely disappearing when Snomax is exposed to temperatures above 35 °C (*SI Appendix*, Fig. S5). Under such conditions, the freezing activity is primarily due to the dimers of class C INs. This is in line with our observation that no loss of cumulative IN concentrations occurs up to 30 °C and that *N*_m_ only decreases beyond 35 °C. We conclude that elevated temperatures impact the stability of the larger, more efficient INP aggregates, while preserving the INP dimers.

However, class C in the heated Snomax displays a broader distribution than in the untreated samples. Previous studies showed that temperatures up to 35 °C do not cause secondary structure changes in INPs (25, 41). Hence, we interpret that the broadening of the peak C arises from a broadening of the intermolecular distances in the dimer. It has been previously shown that variation in the distance between INPs in the dimer as small as 0.1 nm is sufficient to reduce the nucleation temperature to that of the monomer (3). We propose that these small modulations in distance within the dimers, induced by changes in temperature and pH (Fig. 5), are responsible for the wide range of ice nucleating temperatures in the peak C observed in the treated Snomax samples.

Membrane fluidity measurements show that the inherently rigid OM (46) becomes more fluid upon heating (Fig. 5*C* and *SI Appendix*, Fig. S6). This increase is associated with enhanced lateral mobility of membrane components, enabling INPs to diffuse within the more fluid membrane, thus facilitating the disassembly of larger INP aggregates. The finding that enhanced membrane fluidity disintegrates class A INs is supported by our freezing spectra in the absence of ions, at low pH, and in the presence of 2-phenylethanol (PhEtOH). All these conditions are known to increase membrane fluidity (47), and induce similar changes to the distribution of bacterial IN classes (Fig. 5 *A* and *B*). In all spectra, only class C INs prevail, and the additional shift observed e.g., for PhEtOH can be explained by the colligative melting point depression. The minimal temperature dependence of class C INs starkly contrasts with the environmental sensitivity of the larger INP aggregates into which they assemble.

### Dulbecco’s Phosphate-Buffered Saline (DPBS) Buffer Stabilizes and Promotes the Formation of Large Class A Aggregates.

**Fig. 6.**
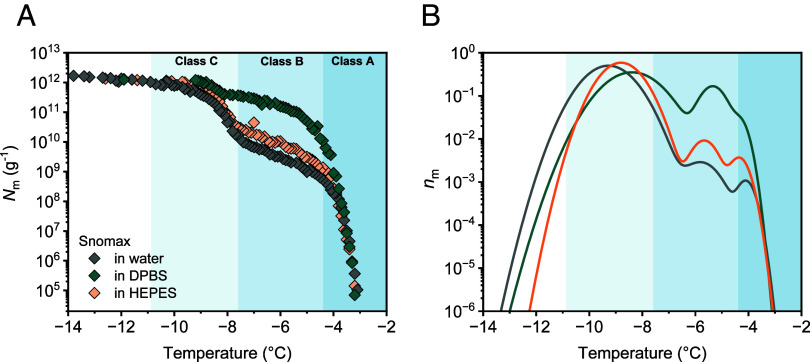
Freezing experiments of aqueous samples containing bacterial INs from Snomax in different buffers. (*A*) Cumulative number of INs per unit mass of Snomax in water (gray). DPBS (green) efficiently promotes larger INP assemblies, whereas HEPES (light orange) exhibits no significant aggregation-enhancing properties, as it only maintains the pH level. (*B*) Normalized distribution functions that represent the corresponding differential freezing spectra *n*_m_(T).

Having identified which parameters decrease class A IN activity, we now address whether it is possible to stabilize and promote the formation of the large class A IN. Fig. 6 shows freezing spectra of Snomax in water, DPBS, and HEPES buffer at pH 7. Strikingly, we find a tremendous enhancement of class A IN activity when measurements were performed in DPBS buffer, which contains sodium and potassium chloride in addition to the Na_2_HPO_4_/KH_2_PO_4_ buffer system. The combined fraction of class A and B increases 200-fold, from 0.1% in water to 19.5% in DPBS. We further observed only a minimal shift toward lower temperatures, despite an expected melting point depression caused by the buffer compounds. This demonstrates that DPBS buffer efficiently promotes larger INP assemblies. Moreover, DBPS endows the bacteria with enhanced stability toward repeated freeze-thaw cycles (*SI Appendix*, Fig. S10).

Maintaining the pH level within a physiological range is critical for the stability of INP multimers (28), as moving to acidic pH levels and approaching the isoelectric point of the INPs prevents the formation of multimers, highlighting that electrostatic interactions between dimer units play a pivotal role in functional INP aggregation. Yet, measurements in HEPES buffer show that the optimal pH range alone is insufficient to enhance INP activity (Fig. 6). Measurements in the presence of HEPES at the same pH as DPBS did not result in enhancement (Fig. 6). DPBS contains cations absent in HEPES, suggesting that there are specific ion effects influencing the stabilization of large INP aggregates. By systematically measuring Snomax in the presence of the different buffer components (*SI Appendix*, Fig. S10*B*), we reveal that the presence of sodium and potassium chloride with the Na_2_KHPO_4_/KH_2_PO_4_ buffer system in DPBS is required to effectively stabilize INP multimers in Snomax. Experiments in NaCl confirm that salt alone has no effect on the class A INs in aqueous solutions, while it shows a clear increase in INP stabilization in the buffer (*SI Appendix*, Fig. S10).

*SI Appendix*, Fig. S11 demonstrates that enhancement effects can be induced in buffers that were originally not showing enhancement through the addition of salts (e.g., adding NaCl to HEPES buffer). We interpret that the resultant concentration of monovalent cations screens the highly negatively charged lipopolysaccharides (LPS) in the OM, strengthening the interactions between the negatively charged proteins (47). We propose that the ion/buffer system limits electrostatic interactions between the predominantly negative LPS and INPs, stabilizes membrane integrity, and thus promotes interactions between INP dimers for aggregation.

## Conclusions

Our analysis of experimental freezing spectra for *P. syringae* and Snomax integrates direct experimental data with numerical modeling (HUB), protein structure prediction (AlphaFold), and CNT for finite-size surfaces (HINT). This comprehensive approach, applied to a range of drop-freezing experiments across various conditions, is a powerful tool for identifying protein aggregates in each IN class of *P. syringae* and understanding their response to environmental factors.

Our results and analyses provide a unifying, quantitative picture of the assembly mechanism of bacterial ice nucleation and the properties of distinct IN classes. By combining results from droplet freezing experiments with stochastic optimization procedures, we identify more than the two commonly discussed class A and class C INs observed in the cumulative freezing spectra of ice-active bacteria. Using the improved structural prediction by AlphaFold of the width and length of the ice-binding site of the bacterial protein combined with nucleation theory calculations of the heterogeneous ice nucleation temperature for finite-size surfaces, we determine the distinct sizes of the different INP aggregates in the bacteria. We confirm that INP dimers are the fundamental unit for aggregation and class C activity. The match between the temperature of the peak for class C and the one deduced -without any adjustment- for the dimer validates this assignment. Moreover, our assignment for the dimer is consistent with the *T*_50_ values and width of INP fibers reported by Hansen et al. (41). Our results further unveil that Class A INs consist of different multimers comprising at least INP hexamers.

Our findings highlight that class A IN activity is achieved by INP aggregates with sizes as small as ~720 kDa, about 25 times smaller than previously proposed sizes of ~19,000 kDa (24). We propose that the formation of the multimers is based on electrostatic interactions between INP dimers, which show a distinctive complementary pattern of outward-facing regions with high charge densities. Our work further resolves the enigma of the originally proposed class B, whose existence we now confirm and ascribed to INP tetramers.

**Fig. 7.**
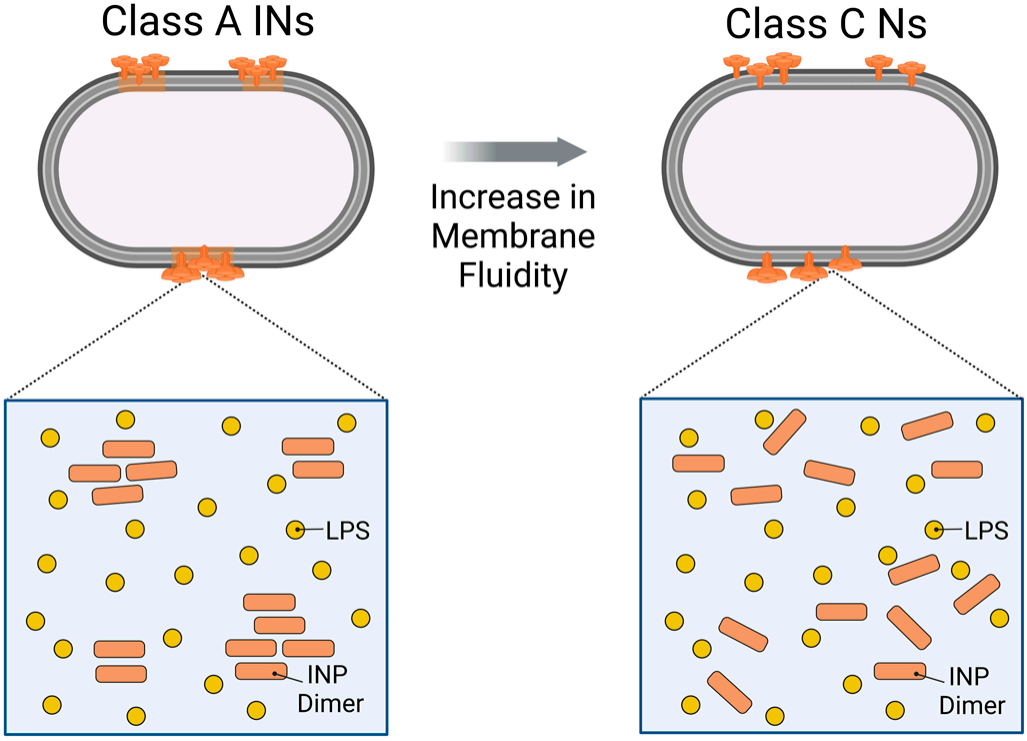
Proposed mechanism of functional INP aggregation in the bacterial OM. Environmental factors such as pH, cosolutes, or temperature can alter membrane fluidity and interprotein interactions, which results in the disassembly of the large functional INP aggregates necessary for ice nucleation activity at high subzero temperatures.

Our systematic alteration of experimental conditions (e.g., pH, temperature, cosolutes) provides conclusive evidence supporting a mechanism where the highest ice nucleation activity in bacteria originates from the formation of functional INP aggregates within the OM as schematically shown in Fig. 7. This is further underlined by a recent study highlighting that class C INs are insensitive to extreme pH from 2 to 11 and heating when the membrane is absent (41). In contrast, moderate pH and temperature changes are sufficient to drastically decrease class A IN activity in the intact membrane (28).

Remarkably, we find that bacterial ice nucleation can be enhanced by stabilizing INP and membrane–protein interactions through carefully balancing external factors (ions, pH, temperature). We demonstrate that the conditions that enhance the population of class A aggregates also protect the bacteria against loss of ice nucleation potency upon freeze-thaw cycles. We conjecture that the enhancement arises from the correct alignment of INPs within the aggregates upon screening of their negative charge. The sensitivity to environmental conditions is consistent with the predictions that even slight (e.g. 0.1 nm) variations of distances between INPs (e.g., due to environmental changes) destabilize the budding ice embryos on the protein surface, dramatically decreasing the ice nucleation temperature (3). Bacteria must exert exquisite control of the distance and alignment of the INP on its OM to freeze ice at temperatures as high as −2 °C (3). Our findings in this work demonstrate that this control can be effectively exerted through environmental variables, broadening the range of uses of bacterial INPs for freezing applications and opening the question of whether these controls are used by the bacteria under physiological conditions. Enhanced stabilization of the INP multimers could further facilitate experiments that resolve the atomistic details of functional INP aggregates in live bacteria and during ice formation. This is essential for obtaining direct evidence of the number of INPs in the functional aggregates, a goal that remains unachieved despite five decades of research on ice nucleation by *P. syringae*.

## Materials and Methods

### Materials.

Pure water was obtained from Millipore Milli-Q® Integral 3 water purification system (Merck Chemicals GmbH, Darmstadt, Germany), autoclaved at 121 °C for 15 min and filtered through a 0.1 µm bottle top filtration unit (VWR International GmbH, Darmstadt, Germany). Snomax® was purchased from SMI Snow Maker AG (Thun, Switzerland) and contains a preparation of inactivated bacteria cells of *P. syringae*. DPBS (without CaCl_2_ and MgCl_2_), HEPES, MOPS, and Laurdan (6-Dodecanoyl-N,N-dimethyl-2-naphthylamine) were purchased from Sigma-Aldrich (Darmstadt, Germany). 2-Phenylethylalcohol was purchased from TCI (Tokyo, Japan). NaOH, NaCl, Na_2_HPO_4_, and KH_2_PO_4_ were purchased from Carl Roth (Karlsruhe, Germany) and KCl from Serva Electrophoresis GmbH (Heidelberg, Germany). The *P. syringae* CiT7 strain was provided by Steven Lindow from the University of California, Berkeley.

### Sample Preparation.

*P. syringae* CiT7 were grown on King B agar for 3 d at 21 °C before assaying. The samples were prepared in pure water with a concentration of 0.1 mg/mL. Snomax samples were prepared in pure water, 0.1 M buffer, and various salt solutions with a concentration of 0.1 mg/mL. Commercial DPBS buffer (Sigma D1408, pH 7) was used as received. MOPS (pH 7) and HEPES buffer (pH 7) were prepared without adding salts and the pH values were adjusted by adding NaOH. Components of DPBS were measured by preparing a 9.57 mM Na_2_HPO_4_/KH_2_PO_4_ solution, a 0.14 M NaCl solution, a 2.68 mM KCl solutions, and combined salt solutions of the same molarities. For dialysis measurements, Snomax samples were dialyzed at 4 °C against pure water for 24 h, and for cosolutes measurements in the presence of 50 mM 2-Phenylethanol.

### TINA Measurements.

Ice nucleation experiments were performed using the high-throughput Twin-plate Ice Nucleation Assay (TINA), which has been described in detail elsewhere (48). In a typical experiment, the investigated IN sample was serially diluted 10-fold by a liquid handling station (epMotion ep5073, Eppendorf, Hamburg, Germany). 96 droplets (droplet volume: 3 μL) per dilution were placed on two 384-well plates and tested with a continuous cooling-rate of 1 °C/min from 0 to −30 °C. The droplet freezing events were detected by two infrared cameras (Seek Thermal Compact XR, Seek Thermal Inc., Santa Barbara, CA). The uncertainty in the temperature of the setup was ± 0.2 °C. The cumulative number of INs was inferred from the obtained fraction of frozen droplets using the Vali formula (49). Experiments were performed at least three times on independent samples. Background freezing of pure water in our system occurred at ~−21 °C.

#### Temperature-dependent measurements.

For freeze-thaw experiments of *P. syringae,* samples of a concentration of 0.1 mg/mL in pure water were serially diluted twofold to create dilutions ranging from 0.1 to 0.5 µg/mL. After being cooled down to −30 °C, the samples were allowed to thaw at room temperature before the next measurement. 15 consecutive freeze-thaw cycles were performed. The same procedure was carried out for freeze-thaw experiments of Snomax in pure water (0.1 mg/mL, 10-fold dilution series, 12 cycles) and in 0.1 M DBPS buffer solution (0.1 mg/mL, 10-fold dilution series, 12 cycles). Snomax samples were prepared in pure water with a concentration of 0.1 mg/mL and heated to temperatures ranging from 20 to 45 °C for 1 h prior to measurement (0.1 mg/mL, 10-fold dilution series).

### Identification of the Ice Nucleating Subpopulations through HUB Analysis.

The HUB method (35) was utilized for the identification and quantification of the subpopulations that constitute the experimental cumulative freezing spectra. This method uses a stochastic optimization technique to extract the underlying distribution of heterogeneous ice nucleation temperatures *P*_u_(*T*) that describes the characteristic freezing temperatures of all INs in a sample. For this, the HUB-backward code available as a Python code (https://github.com/Molinero-Group/underlying-distribution) was used to compute the differential freezing spectra *n*_m_(*T*), representing *P*_u_(*T*), from the cumulative freezing spectra *N*_m_(*T*) obtained from TINA experiments. *P*_u_(*T*) is assumed to be a linear combination of normalized Gaussian distribution functions *P*_i_(*T*) that represents a distinct number of subpopulations *p* of the weights *c*_i_ that give $\sum_{i=1}^{p}c_{i}=1$. Each subpopulation *P*_i_(*T*) is further characterized by its characteristic freezing temperature mode *T*_mode,i_ and the spread of the temperature distribution *s*_i_. The experimentally obtained *N*_m_(*T*) is interpolated through a spline and smoothed with a Savitzky–Golay filter of first polynomial order with a default value of 3 for the length of the filter window. For Snomax, data are interpolated with a number of 100 equally spaced points and for bacterial samples from *P. syringae* with 500 points. The mean squared error MSE defines the accuracy of the determined set of parameters for the distribution function. For further analysis, optimized results with the lowest MSE were selected.

### Prediction of the Protein Structures with AlphaFold.

AlphaFold (V2.0.1) and (V.2.3.2) were used for structure predictions with the required databases downloaded from the AF2 GitHub repository (43). The best ranked AlphaFold monomer model of the INP using V.2.01 contained a kink close to residue D555 (40). The newer AlphaFold version predicts a straighter INP β-helix. However other ranked models constructed by V.2.3.2 also exhibited kinks/twists (*SI Appendix*, Fig. S4). The AlphaFold predictions of the INP dimers and multimeric aggregates exhibited twisted and kinked structures (*SI Appendix*, Fig. S4) that do not match the observed high ice nucleation temperatures. All models were constructed using the same settings except the maximal template date was set as 09-15-2021 for the old model, and 08-30-2023 for the newer models.

### Prediction of the Ice Nucleation Temperatures of INP Aggregates.

The HINT algorithm accurately implements numerical CNT to predict the heterogeneous nucleation temperature of ice on finite-sized surfaces using thermodynamic and dynamic data from water, as well as the binding free energy of the IN to ice Δγ_bind_ (3, 50). With that data, HINT computes the free energy barriers for ice nucleation and the prefactor for the nucleation rate, using as reference that the experimental ice nucleation rate *J*_exp_ = 10^5^ cm^−3^ s^−1^ for microliter droplets cooled at 1 °C/min (51–53). We start by assuming that *P. syringae’s* INP binds ice as strong as ice itself, i.e., Δγ_bind_ = −2 γ_ice–liquid_, as demonstrated in ref. 3. Additionally, we consider a line tension of *τ* = 10 pN. The IN surface area is assumed to be rectangular, with a width *W* = 3.4 nm and length *L* = 30 nm, consistent with the structure predicted by AlphaFold (40). We assume that functional aggregates with *n* INP have width *W*_n_ = *n* × 3.4 nm and length *L* = 30 nm, consistent with the model of assembly we propose in this study.

Using the HINT code, we calculate the free energy cost Δ*G* for various configurations by adjusting the number of water molecules N* and the contact angle *θ* of the ice nucleus on the nucleating surface (3). This allows us to identify the optimal size for ice growth with the lowest energy cost, determined by the smallest Δ*G* for a specific number of water molecules. From this set, we identify the ice nucleation barrier ΔG*(*T*), which corresponds to the highest value of Δ*(*T*) within a temperature range from *T_m_* to *T_hom_*, with a resolution of 0.1 K. We continue this computation until the calculated ΔG*(*T*) matches the value derived from the homogeneous nucleation rate (3).

### Membrane Fluidity Measurement.

For the measurement of membrane fluidity, a 10 mM stock solution of the membrane dye Laurdan in DMF was prepared. Aqueous samples of Snomax were prepared at a concentration of 1 mg/mL. Laurdan solution was added to a final concentration of 40 µM, and the samples were stirred for 1 h at 500 rpm. The stained samples were washed twice to remove excess dye, by centrifugation at 15,000× g for and after the final washing step, the samples were subjected to the desired temperature using a thermomixer. Snomax samples containing 2-phenylethanol (50 mM) were equilibrated at 21 °C. For temperature-dependent measurements, Snomax samples were treated at different temperatures for 15 min. The temperature series started at 10 °C and proceeded in 5 °C intervals up to 50 °C. Fluorescence measurements (TIDAS FL3095 SL, J&M, Essingen, Germany) were performed using an excitation wavelength of 350 nm, and fluorescence emission was recorded from 370 to 800 nm. The integration time was set to 10,000 ms and emission spectra were obtained by averaging three measurements. To determine the membrane fluidity, the spectra were normalized and the generalized polarization (*GP*) was calculated using

$$GP=\frac{I_{440\mathrm{nm}}-I_{490\mathrm{nm}}}{I_{440\mathrm{nm}}+I_{490\mathrm{nm}}},$$

where *I*_440 nm_ is the fluorescence emission intensity at 440 nm and *I*_490 nm_ is the intensity at 490 nm. High *GP* values correspond to low membrane fluidity and low values to high membrane fluidity.

## Supplementary Material

Appendix 01 (PDF)

Dataset S01 (XLSX)

## Data Availability

All study data are included in the article and/or supporting information.
