# Supplementary material for: Hierarchical assembly and environmental enhancement of bacterial ice nucleators
Source: Proc Natl Acad Sci U S A. 2024 Oct 17;121(43):e2409283121. doi: 10.1073/pnas.2409283121 (PMC11513900; doi:10.1073/pnas.2409283121)
Supplement: Supplementary file 1 — Appendix 01 (PDF) [file pnas.2409283121.sapp.pdf]

Supporting Information for

Hierarchical Assembly and Environmental Enhancement of Bacterial Ice Nucleators

Galit Renzer[a,1], Ingrid de Almeida Ribeiro[b,1], Hao-Bo Guo[c], Janine Fröhlich-Nowoisky[d], Rajiv Berry[c], Mischa Bonn[a], Valeria Molinero[b,2], and Konrad Meister[a,e,2]

[a]Max Planck Institute for Polymer Research, 55128 Mainz, Germany

[b]Department of Chemistry, The University of Utah, 84112-0850 Salt Lake City, UT, USA

[c]Air Force Research Laboratory, Wright-Patterson Air Force Base, Dayton, OH, 45433, USA

[d]Max Planck Institute for Chemistry, 55128 Mainz, Germany

[e]Department of Chemistry and Biochemistry, Boise State University, 83725 Boise, ID, USA

[1]G.R. and I.d.A.R. contributed equally to this work

[2]To whom correspondence may be addressed. Email: valeria.molinero@utah.edu

or meisterk@mpip-mainz.mpg.de .

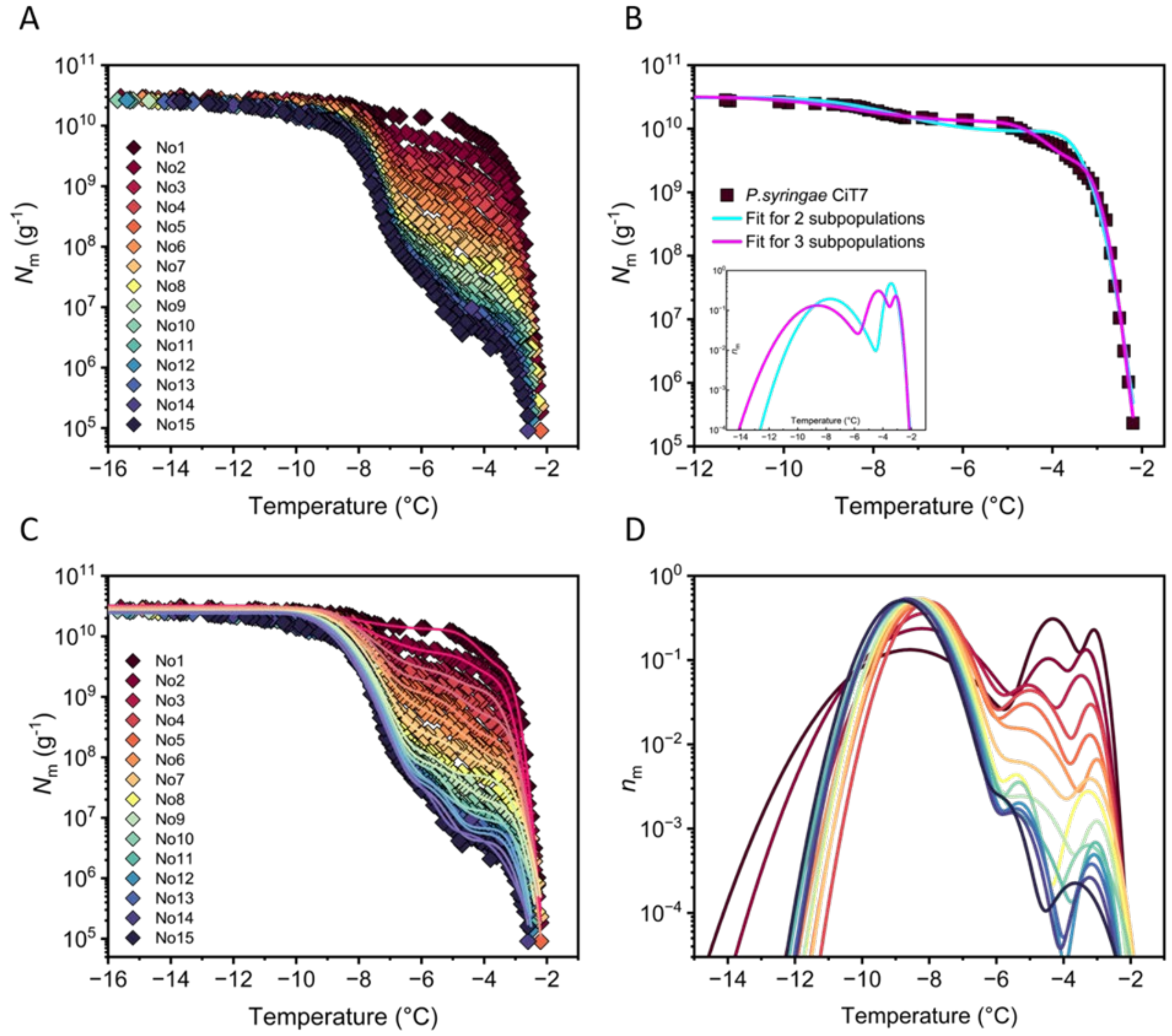

Fig. S1. **Repetitive freeze-thaw cycles of aqueous samples containing bacterial INs from *P. syringae* and corresponding results of the HUB analysis.** (A) Cumulative number of INs per unit mass of *P. syringae* (N_m) of all conducted freeze-thaw cycles. (B) Optimized solutions for the initial freezing spectrum by assuming linear combinations of two (cyan) and three (magenta) Gaussian subpopulations obtained through the HUB method (1). Inset shows the corresponding normalized distribution functions. (C) Optimized solutions of all freezing spectra obtained in the experiment by assuming that the differential spectrum is a linear combination of three Gaussian subpopulations. (D) Normalized distribution functions that represent the corresponding differential spectra n_m(T).

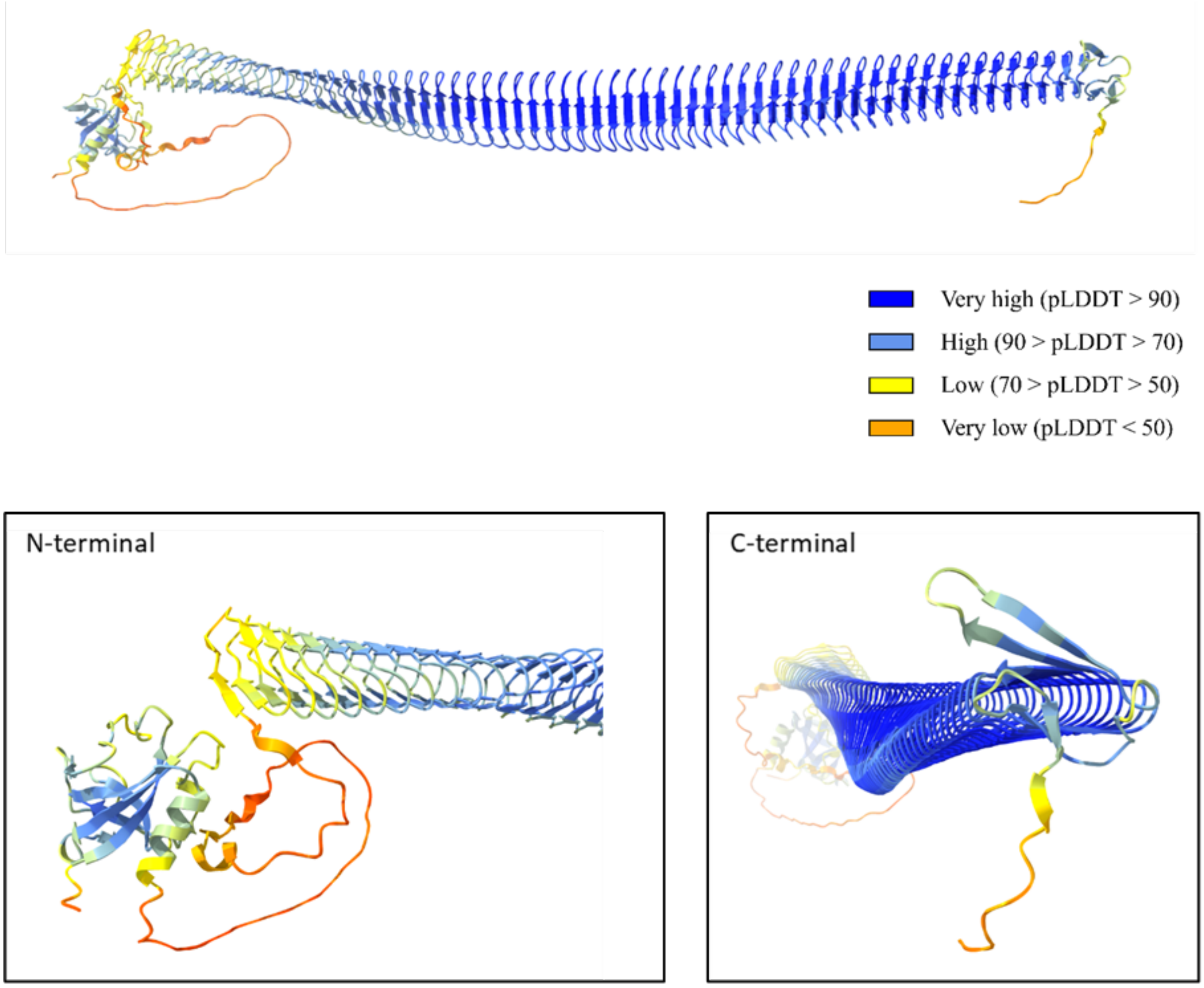

Fig. S2. **Predicted AlphaFold model of the INP structure of *P. syringae*.** The accuracy of the structural prediction is reflected in the color-coded confidence score named pLDDT (predicted value of the Local Distance Difference Test) ranging from 0 -100. pLDDT > 90 indicates prediction with extremely high confidence and values between 90 > pLDDT > 70 high confidence, whereas 70 > pLDDT > 50 is classified as low confidence and pLDDT < 50 is extremely low. Regions predicted with lower confidence by AlphaFold are more dynamic (2). The calculations were performed with Alphafold version 2.3.2 (3).

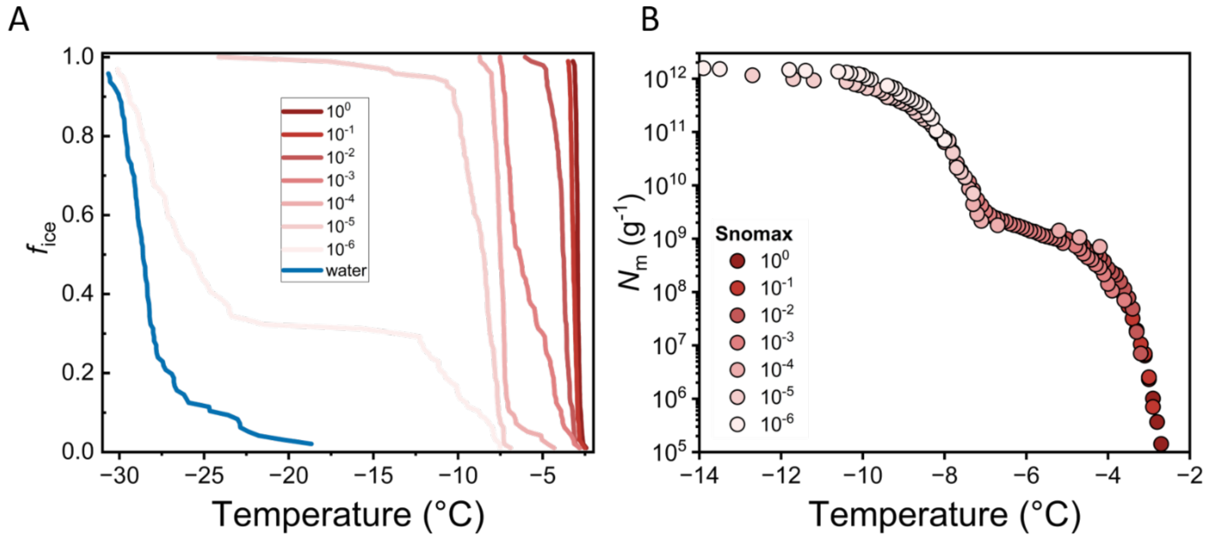

Fig. S3. Freezing experiments of aqueous solutions of Snomax containing bacterial ice nucleators from *P. syringae*. (A) Fraction of frozen droplets (f_{ice}) vs temperature for the dilution series of Snomax in pure water with an initial concentration of 0.1 mg/mL. (B) Cumulative number of INs per unit mass (N_m).

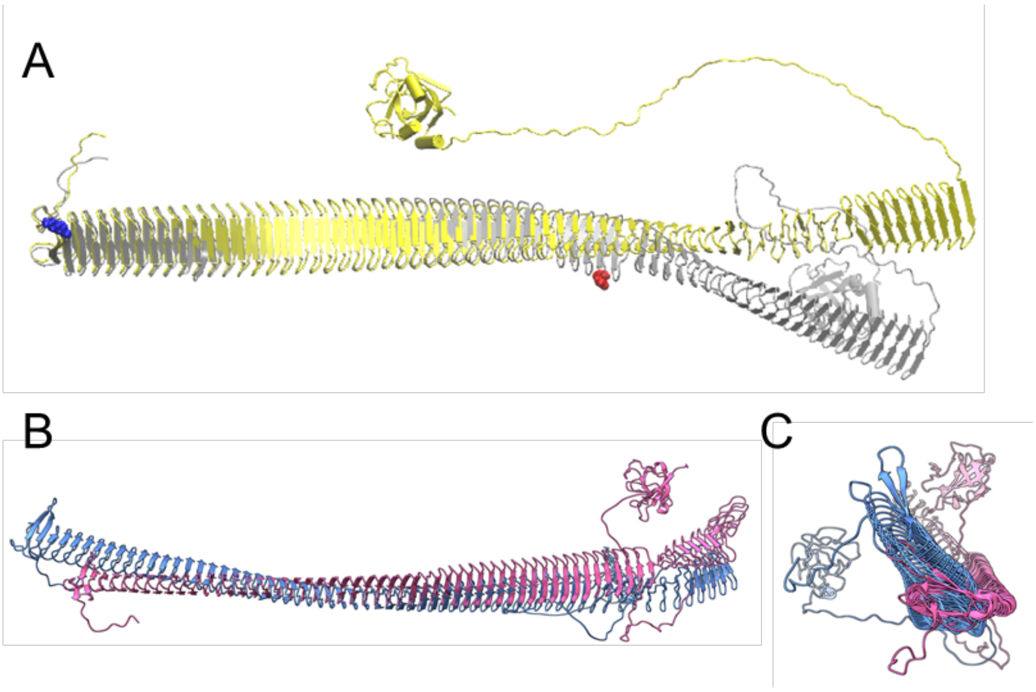

Fig. S4. Predicted AlphaFold model of INP structures of *P. syringae*. (A) Overlay of the predicted INP structure of *P. syringae* using an older (V.2.0.1, grey) and newer (V.2.3.2, yellow) version of Alphafold (2,3). The proteins are aligned using the alpha carbons D555 (red spheres) to K1158 (blue spheres). (B) Alphafold V.2.3.2 (3) predicts that the dimer (monomers displayed in pink and blue) and higher multimeric aggregate (not shown) have twisted, amyloid-like structures that bury the ice-nucleating surfaces. (C) Frontal-view of the predicted INP dimer structure using Alphafold V.2.3.2 (3).

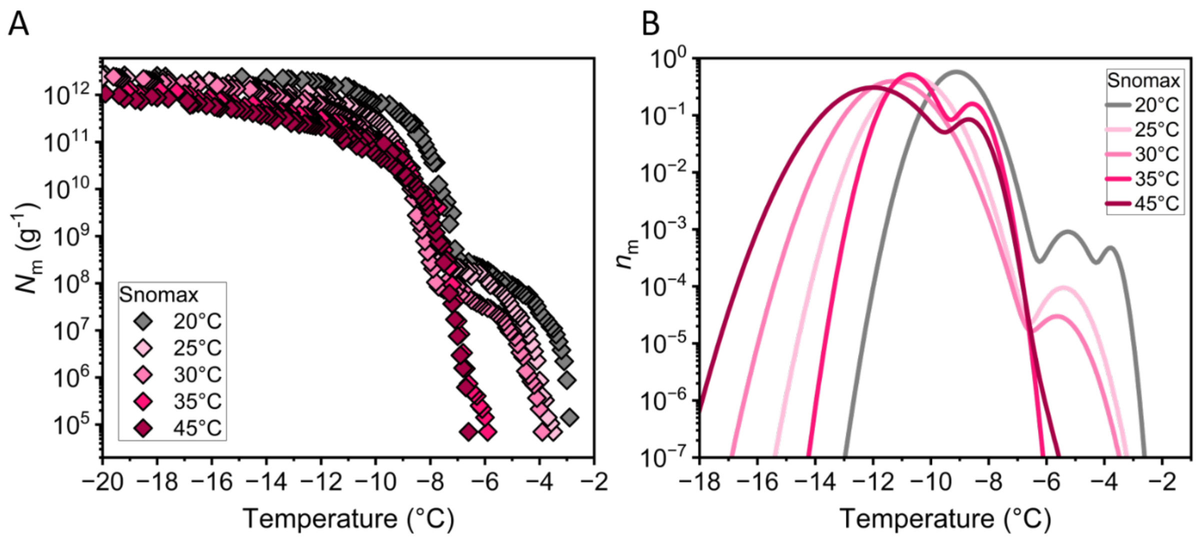

Fig. S5. Freezing experiments of aqueous solutions of Snomax containing bacterial ice nucleators from *P. syringae* as a function of temperature. (A) Cumulative freezing spectra of a dilution series of Snomax in pure water with an initial concentration of 0.1 mg/mL after heating to temperatures ranging from 20 to 45°C and (B) the corresponding normalized distribution functions obtained through the HUB method.

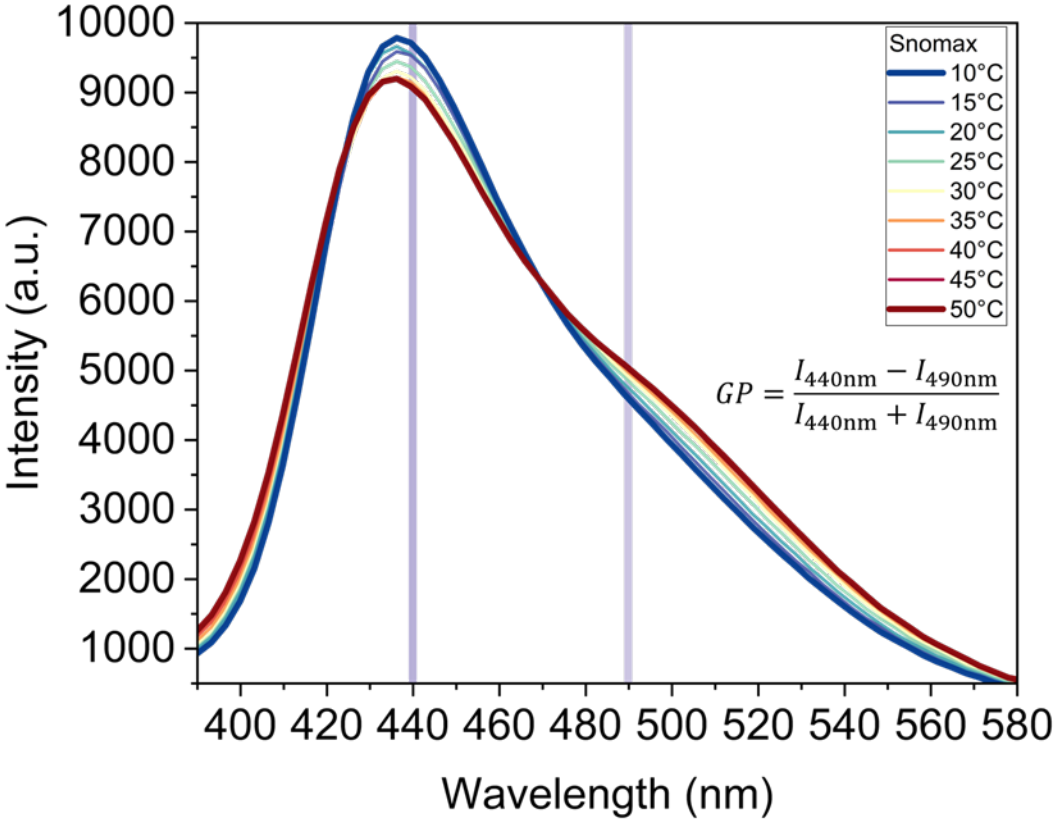

Fig. S6. Membrane fluidity measurements of aqueous solutions of Snomax stained with the membrane dye Laurdan at temperatures from 10 to 50°C. The generalized polarisation (GP) is a measure for the fluidity of the outer membrane. GP values are obtained by using the fluorescence intensity of Laurdan at 440 and 490 nm.

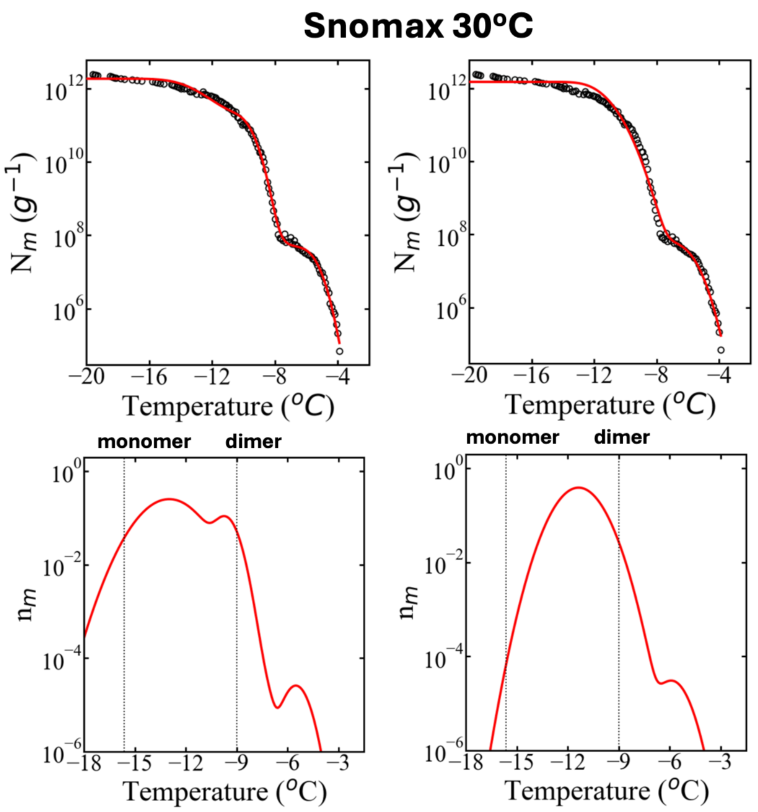

Fig. S7. Subpopulation analysis of freezing experiments of aqueous samples containing Snomax heated to 30ºC. Upper panels show the cumulative number of INs per unit mass of Snomax (black circles). Red continuous lines are the fits done using the HUB-backward code using 3 (left) and 2 (right) subpopulations. Lower panels show the normalized distribution functions that represent the corresponding differential freezing spectra represented with 3 (left) and 2 (right) subpopulations. The parameters of the fits are shown in Table S3. The black dotted lines indicate the freezing temperature of the INP monomer and dimer obtained through the HINT code with the protein ice-binding surface of the protein structure predicted by AlphaFold.

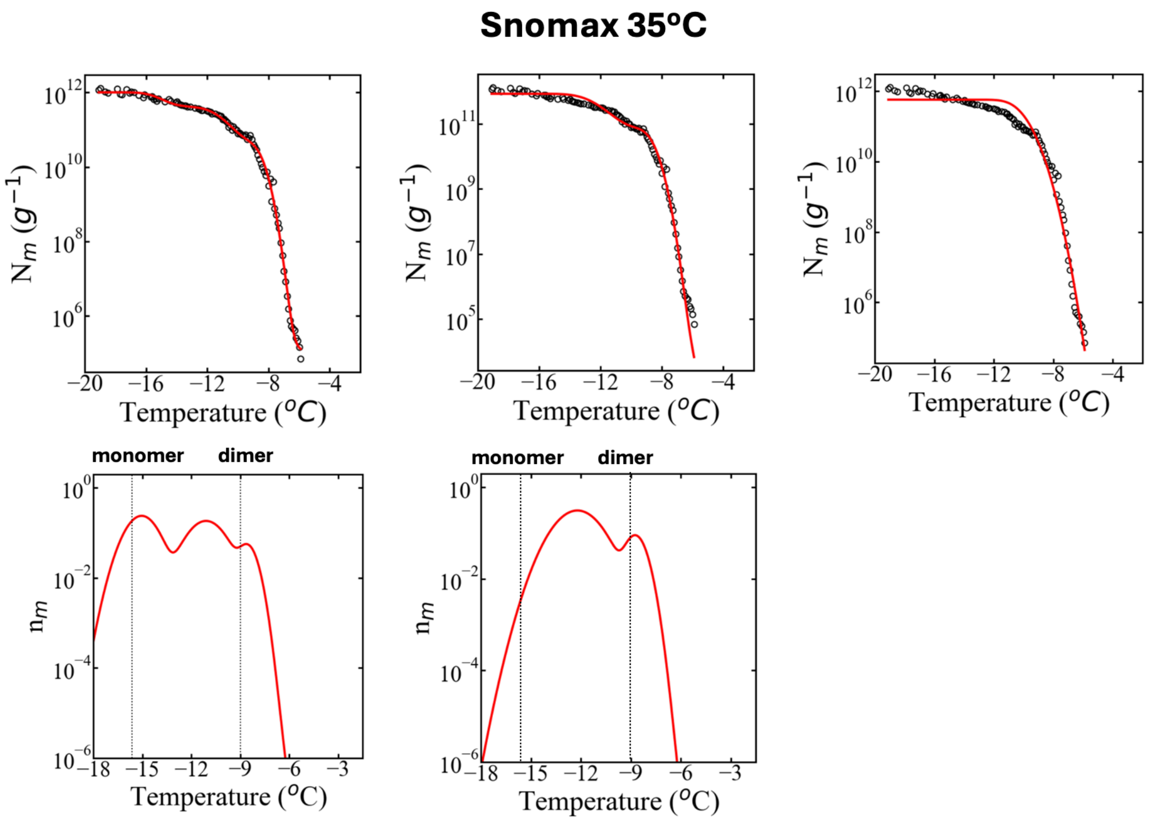

Fig. S8. Subpopulation analysis of freezing experiments of aqueous samples containing Snomax heated to 35ºC. Upper panels show the cumulative number of INs per unit mass of Snomax (black circles). Red continuous lines are the fits done using the HUB-backward code using 3 (left), 2 (middle) and 1 (right) subpopulations. Lower panels show the normalized distribution functions that represent the corresponding differential freezing spectra represented with 3 (left), and 2 (right) subpopulations. The parameters of the fits are shown in Table S3. The black dotted lines indicate the freezing temperature of the INP monomer and dimer obtained through the HINT code with the protein ice-binding surface of the protein structure predicted by AlphaFold.

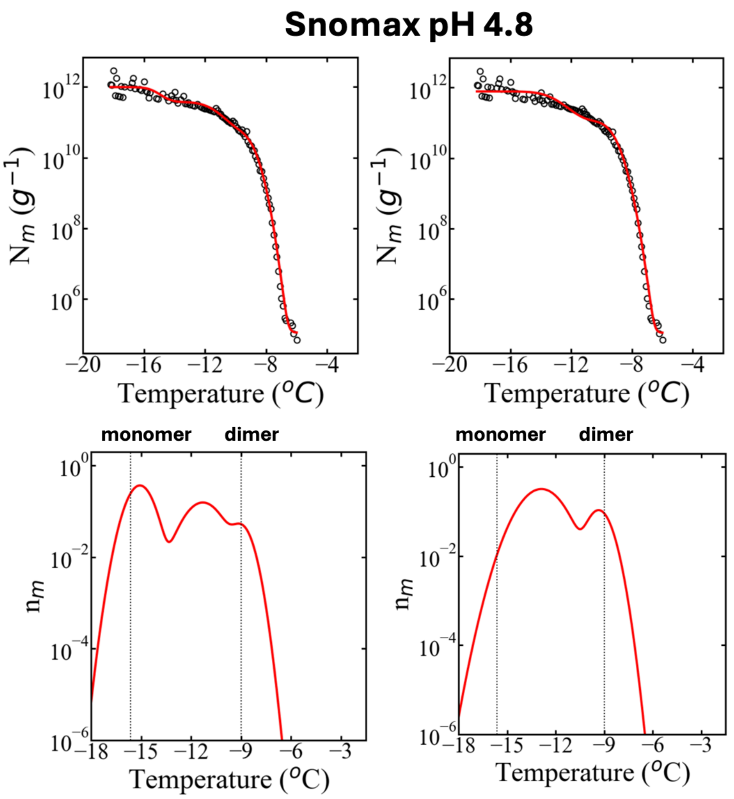

Fig. S9. Subpopulation analysis of freezing experiments of aqueous samples containing bacterial IN from Snomax after lowering the solution pH. Upper panels show the cumulative number of INs per unit mass of Snomax (black circles). Red continuous lines are the fits done using the HUB-backward code using 3 (left) and 2 (right) subpopulations. Lower panels show the normalized distribution functions that represent the corresponding differential freezing spectra represented with 3 (left), and 2 (right) subpopulations. The parameters of the fits are shown in Table S3. The black dotted lines indicate the freezing temperature of the INP monomer and dimer obtained through the HINT code with the protein ice-binding surface of the protein structure predicted by AlphaFold.

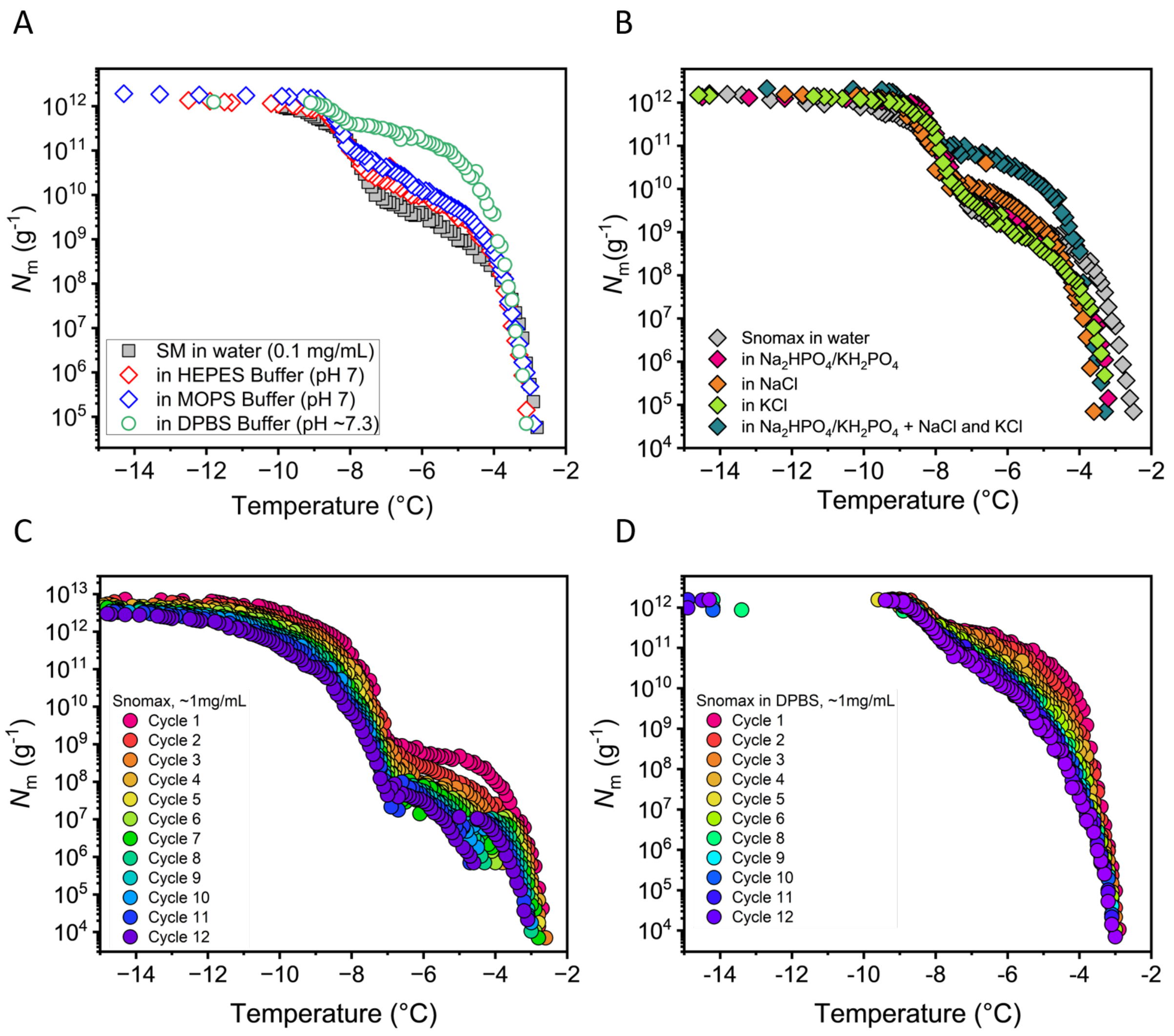

Fig. S10. Freezing experiments of solutions of Snomax containing bacterial ice nucleators from *P. syringae* in different solutions. (A) Cumulative freezing spectra of a dilution series of Snomax in water and in different buffer at pH 7. (B) Cumulative freezing spectra of a dilution series of Snomax in the different buffer components of DPBS. Enhancement of activity appears only after NaCl and KCl addition to the phosphate buffer system Na2HPO4/KH2PO4 (dark green). (C) Freeze-thaw cycles of an aqueous solution of Snomax and (D) of a solution of Snomax in DPBS buffer.

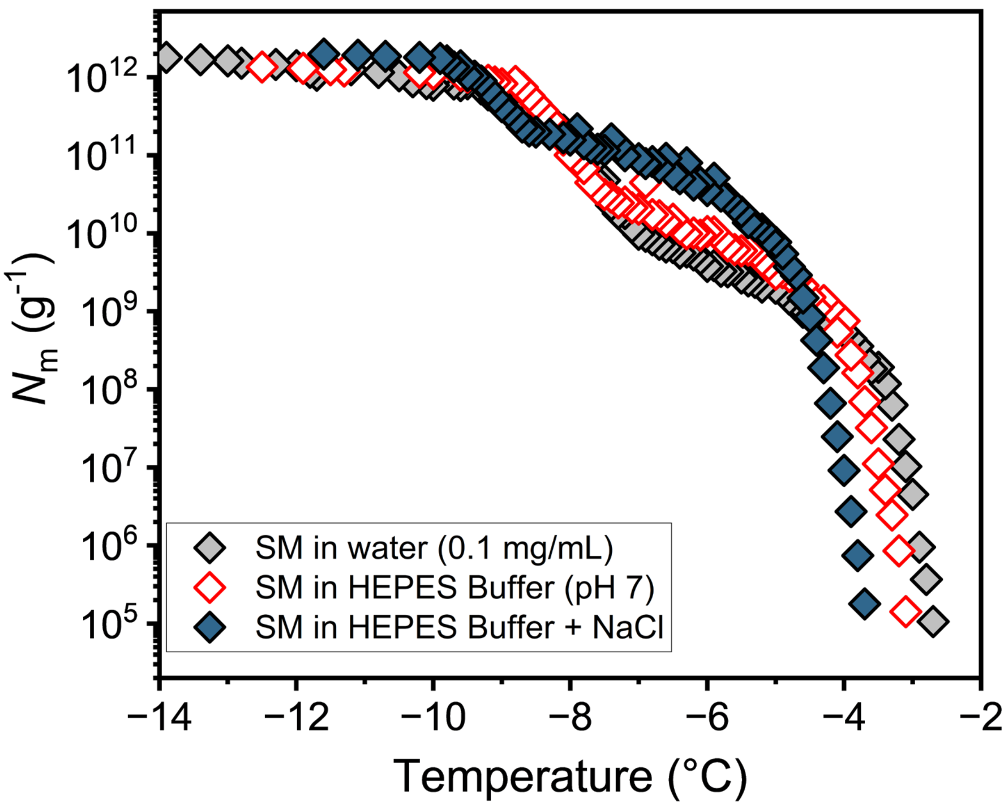

Fig. S11. Freezing experiments of Snomax solutions containing bacterial ice nucleators from *P. syringae* in HEPES buffer (red) and HEPES buffer with 10 mg/mL NaCl (blue). Enhancement of activity appears after the addition of the salt to the buffer.

Table S1. Parameter of the normalized distribution functions of the freezing spectra after repetitive freeze-thaw cycles obtained through the HUB method (1) using three Gaussian subpopulations. T_{mode} is the mode (maximum) of the Gaussian distribution of heterogeneous ice nucleation temperatures of the corresponding subpopulation, s is the spread the distribution of the subpopulations, and c the fraction of the subpopulation. The mean squared error (MSE) describes the quality of the found optimized solution for the cumulative spectra obtained with the HUB analysis relative to the experimental cumulative spectra.

**Cycle**	$T_{mode,1}$ (°C)	s_1	c_1	$T_{mode,2}$ (°C)	s_2	c_2	$T_{mode,3}$ (°C)	s_3	c_3	**MSE**
**1**	-8.57	1.46	0.4878	-4.32	0.50	0.3867	-3.08	0.23	0.1255	0.001
**2**	-8.21	1.32	0.7804	-4.43	0.51	0.1277	-3.30	0.3	0.0919	0.0017
**3**	-8.10	0.96	0.8713	-5.00	0.56	0.0683	-3.47	0.37	0.0604	0.0018
**4**	-8.02	0.73	0.9051	-5.00	0.68	0.0747	-3.18	0.28	0.0202	0.0032
**5**	-8.16	0.77	0.9415	-5.10	0.64	0.04867	-3.20	0.31	0.00983	0.0029
**6**	-8.27	0.83	0.9647	-5.20	0.84	0.0313	-2.99	0.26	0.0040	0.003
**7**	-8.26	0.73	0.9834	-5.31	0.86	0.0128	-3.17	0.40	0.0038	0.0044
**8**	-8.39	0.75	0.9931	-5.37	0.38	0.003921	-3.27	0.43	0.002979	0.0047
**9**	-8.46	0.74	0.9944	-5.18	0.78	0.00469	-3.00	0.32	0.00091	0.0055
**10**	-8.58	0.78	0.9960	-5.31	0.37	0.0032	-3.26	0.50	0.0008	0.0045
**11**	-8.65	0.80	0.9966	-5.58	0.78	0.00288	-3.05	0.30	0.00052	0.005
**12**	-8.62	0.77	0.9975	-5.30	0.42	0.00208	-3.09	0.31	0.00042	0.004
**13**	-8.81	0.78	0.9975	-5.80	0.43	0.0022	-3.65	0.60	0.0003	0.0053
**14**	-8.66	0.75	0.9980	-5.47	0.46	0.00173	-3.23	0.38	0.00027	0.0039
**15**	-8.81	0.78	0.9975	-5.80	0.43	0.00219	-3.65	0.60	0.00031	0.0053

Table S2. Predicted ice nucleation temperatures as a function of the number of protein monomers in the INP aggregates. The ice nucleation temperatures are predicted by classical nucleation theory using the HINT algorithm and are presented as a function of the number of INPs, N_{INP}, from *P. syringae*. The analysis considers rectangular surfaces formed by parallel-aligned INP monomers, assuming a monomer with ice-binding surface of width of 3.4 nm and length 30 nm, as predicted by Alphafold.

T_{het} (°C)	N_{INP}
-15.65	1
-9	2
-6.52	3
-5.18	4
-4.43	5
-4.1	6
-3.79	8
-3.3	12
-2.65	16

Table S3. Parameters of the normalized distribution functions of the freezing spectra of heat- and pH-treated Snomax represented with 3, 2 or 1 subpopulations. T_{mode} is the mode (maximum) of the Gaussian distribution of heterogeneous ice nucleation temperatures of the corresponding subpopulation, *s* is the spread the distribution of the subpopulations, and *c* the fraction of the subpopulation. The mean squared error (MSE) describes the quality of the found optimized solution for the cumulative spectra obtained with the HUB analysis relative to the experimental cumulative spectra.

**Label**	$T_{mode,1}$ (°C)	s_1	c_1	$T_{mode,2}$ (°C)	s_2	c_2	$T_{mode,3}$ (°C)	s_3	c_3	**MSE**
**pH 4.8, 3 subpopulations**	-15.07	0.63	0.58	-11.29	0.92	0.36	-8.99	0.52	0.0599	0.006
**pH 4.8, 2 subpopulations**	-12.88	1.05	0.842	-9.33	0.59	0.158				0.014
**Snomax 35°C, 3 subpopulations**	-15.03	0.83	0.487	-11.12	0.99	0.452	-8.54	0.48	0.061	0.006
**Snomax 35°C, 2 subpopulations**	-12.21	1.13	0.887	-8.73	0.52	0.113				0.016
**Snomax 35°C, 1 subpopulation**	-10.16	0.83								0.07
**Snomax 30°C, 3 subpopulations**	-12.98	1.36	0.864	-9.66	0.56	0.135	-5.51	0.58	$3.8\ 10^{-5}$	0.007
**Snomax 30°C, 2 subpopulations**	-11.36	1.02	0.999	-5.89	0.73	$5.6\ 10^{-5}$				0.03

References:

1. I. de Almeida Ribeiro, K. Meister, V. Molinero, HUB: a method to model and extract the distribution of ice nucleation temperatures from drop-freezing experiments. *Atmos. Chem. Phys.* **23**, 5623–5639 (2023).

2. H.-B. Guo, *et al.*, AlphaFold2 models indicate that protein sequence determines both structure and dynamics. *Sci. Rep.* **12**, 10696 (2022).

3. J. Jumper, *et al.*, Highly accurate protein structure prediction with AlphaFold. *Nature* **596**, 583–589 (2021).
